# Supplementary material for: Comparative multiomics analysis of cell physiological state after culture in a basket bioreactor
Source: Sci Rep. 2022 Nov 23;12:20161. doi: 10.1038/s41598-022-24687-4 (PMC9686226; doi:10.1038/s41598-022-24687-4)
Supplement: Supplementary file 1 — Supplementary Information 1. [file 41598_2022_24687_MOESM1_ESM.zip › raw data/Transcriptomic raw data/7.AS/2.dasplot/TE1vsTC1/RI/10_DALRD3_NW_023666041.1_156665588_156665770_+@NW_023666041.1_156665588_156665956_+@NW_023666041.1_156665843_156665956_+.pdf]

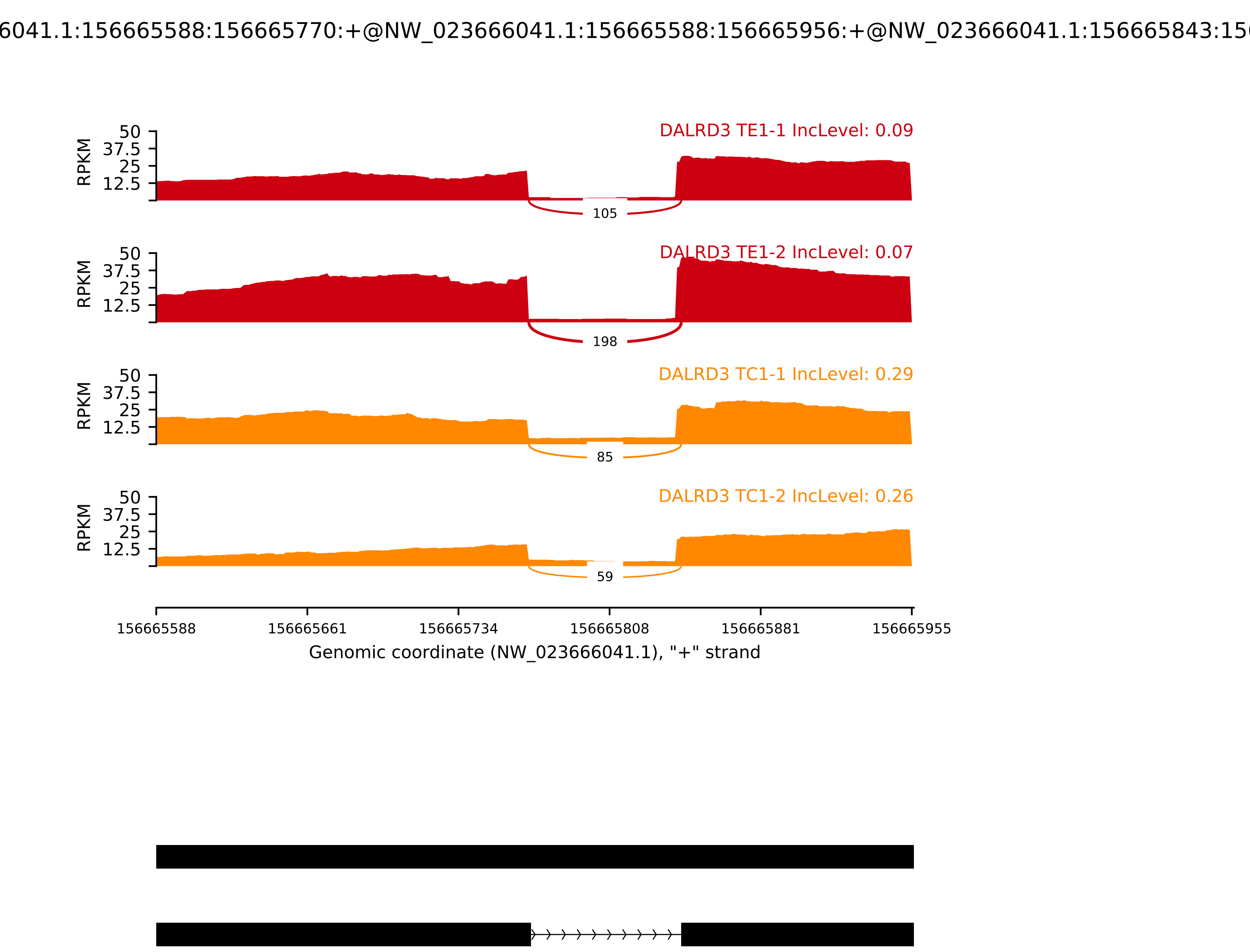
6041.1:156665588:156665770:+@NW_023666041.1:156665588:156665956:+@NW_023666041.1:156665843:15[illegible]
DALRD3 TE1-1 IncLevel: 0.09
105
DALRD3 TE1-2 IncLevel: 0.07
198
DALRD3 TC1-1 IncLevel: 0.29
85
DALRD3 TC1-2 IncLevel: 0.26
59
RPKM
50
37.5
25
12.5
156665588
156665661
156665734
156665808
156665881
156665955
Genomic coordinate (NW_023666041.1), "+" strand
